# ACUTE CARE UTILIZATION IN OLDER ADULTS LIVING UNDIAGNOSED OR UNAWARE OF DEMENTIA

**DOI:** 10.1093/geroni/igz038.2707

**Published:** 2019-11-08

**Authors:** Halima Amjad, David L Roth, Jin Huang, Jennifer L Wolff, Quincy M Samus

**Affiliations:** 1 Johns Hopkins University School of Medicine, Baltimore, Maryland, United States; 2 Johns Hopkins University, Maryland, United States; 3 The Johns Hopkins University, Baltimore, Maryland, United States

## Abstract

Most individuals with dementia are undiagnosed or they/their families are unaware of the diagnosis. Implications of dementia diagnosis and awareness are poorly understood. Our objective was to determine whether undiagnosed dementia or unawareness increases risk of hospitalization or emergency department (ED) visits, outcomes with recognized risk in diagnosed dementia. We linked National Health and Aging Trends Study (NHATS) data to fee-for-service Medicare claims for 4,311 community-living participants in the nationally representative cohort. We assessed probable versus no dementia using validated NHATS dementia criteria, undiagnosed versus diagnosed using Medicare claims, and aware versus unaware using NHATS self or proxy report of diagnosis. Cox proportional hazards models evaluated hospitalization and ED visit risk by time-varying dementia diagnosis and awareness status, adjusting for sociodemographic characteristics, functional impairment, medical comorbidities, and prior hospitalization. Compared to no dementia, persons with dementia who were unaware but diagnosed had greater risk of hospitalization (HR 1.66, 95% CI 1.26-2.19) and ED visits (HR 1.63, 95% CI 1.28-2.08). Persons unaware but diagnosed also had greater risk compared to persons aware and diagnosed (hospitalization HR 1.34, 95% CI 0.98-1.82; ED HR 1.38, 95% CI 1.05-1.83). Persons with undiagnosed dementia demonstrated hospitalization risk similar to persons with no dementia (HR 1.02, 95% CI 0.79-1.31) and similar or potentially lower than persons aware and diagnosed (HR 0.82, 95% CI 0.61-1.10); ED visit findings were similar. Results suggest that being unaware of dementia diagnosis may affect healthcare utilization. Strategies to improve communication and understanding of dementia could potentially reduce hospitalizations and ED visits.

